# Robotic versus Endoscopic Thyroidectomy for Thyroid Cancers: A Multi-Institutional Analysis of Early Postoperative Outcomes and Surgical Learning Curves

**DOI:** 10.1155/2012/734541

**Published:** 2012-11-26

**Authors:** Jandee Lee, Jong Ho Yun, Un Jong Choi, Sang-Wook Kang, Jong Ju Jeong, Woong Youn Chung

**Affiliations:** ^1^Department of Surgery, Eulji University College of Medicine, Seoul, Republic of Korea; ^2^Department of Surgery, Asan Medical Center, Seoul, Republic of Korea; ^3^Department of Surgery, Wonkwang University School of Medicine, Iksan, Republic of Korea; ^4^Department of Surgery, Yonsei University College of Medicine, 134 Shinchon-dong, Seodaemun-ku, Seoul 120-752, Republic of Korea

## Abstract

Robotic thyroidectomy is an emerging technique with postoperative outcomes that are at least comparable to those of conventional endoscopic thyroidectomy, with some end-points appearing superior. Our multicenter series represents the largest comparison of robotic and endoscopic thyroidectomy to date, with results suggesting a comparable robot technology we used that could overcome some of the technical limitations associated with conventional endoscopic procedures, with reduced operation times and increased lymph node retrieval. Moreover, we found that the learning curve for robotic thyroidectomy was shorter than that for endoscopic thyroidectomy.

## 1. Introduction

Endoscopic surgical techniques for thyroid cancer surgery can benefit patients by eliminating the anterior neck incision utilized in the traditional open approach. In addition to superior cosmetic results, endoscopic thyroidectomy can reduce postoperative pain and discomfort, shorten hospital stay, and enhance postoperative recovery [1–4]. Despite these advantages, however, endoscopic thyroidectomy has technical limitations, including the use of straight, rigid endoscopic instruments without articulation and a 2Dimensional (2D) view. 

The recent introduction of the da Vinci robot surgical system may be a major improvement in extracervical approaches for thyroid surgery and may be more ergonomic for surgeons than the endoscopic approach [5–7]. Among the advantages of the da Vinci robot are improved visualization via a 3D view, magnification, a tremor-filtering system, and instrument flexibility. To date, however, few studies have compared the postoperative outcomes in patients undergoing robotic and endoscopic thyroid surgery [8–11].

At present, endoscopic techniques are regarded as too time consuming and technically demanding to be adopted on a large-scale. The learning curve for endoscopic thyroidectomy performed by skilled endocrine surgeons has been estimated to be approximately 60 patients [12]. Use of a robot in thyroid surgery may shorten the learning curve, by providing a broader view of the surgical field and easier access to deep and narrow spaces through the use of multiarticulated instruments. We have previously reported the results of a multicenter study of learning curves for robotic thyroidectomy, based on a scientific analysis of a range of perioperative parameters [13]. However, there have been few comparisons of learning curves for robotic and endoscopic thyroidectomies.

At present, the benefits of robotic thyroidectomy relative to endoscopic thyroidectomy, as determined by oncologic and functional outcomes, have not been fully clarified. Moreover, there have been no multicenter studies comparing learning curves for these two methods. We therefore compared the operative outcomes and surgical learning curves of robotic and endoscopic thyroidectomy in patients with differentiated thyroid carcinoma (DTC).

## 2. Materials and Methods

### 2.1. Study Patients

This comparative, multicenter study evaluated patients who underwent robotic or endoscopic thyroidectomy at three large-volume centers with considerable experience in thyroid cancer surgery. Clinical and pathological data were collected retrospectively at each institution and entered into a dedicated database for analysis. Between November 2001 and June 2010, 2,612 patients with DTC underwent thyroidectomies at three centers, with all operations performed by four surgeons. Of these patients, 1796 underwent robotic thyroidectomy and 843 underwent conventional endoscopic thyroidectomy. At the time of surgery there was no intention to compare the two procedures. This study was approved by the institutional review boards (IRBs).

At all institutions, perioperative workup included physical examination, high-resolution ultrasonography (US), and neck computed tomography (CT) and/or neck magnetic resonance imaging (MRI); preoperative staging US and neck CT (or MRI) was utilized to evaluate the degree of tumor invasion, including tumor size, extrathyroidal invasion, tumor infiltration of adjacent structures, and nodal involvement. Sonographic findings, such as loss of an echogenic thyroid capsule at the contact site of the primary tumor or contact with an adjacent thyroid capsule along more than 25% of the boundary of a tumor, were considered indicative of extrathyroidal extension [14].

In accordance with American Thyroid association (ATA) guidelines, a less than total thyroidectomy was performed in patients <45 years old, with a single lesion <1 cm in size, no definitive evidence of extrathyroidal invasion or lymph node metastasis, no personal history of radiation therapy to the head or neck, and no first-degree family history of DTC [15]. A total thyroidectomy was performed in patients with multiple or bilateral lesions, or if definite extrathyroidal invasion was discovered during surgery. All patients underwent prophylactic ipsilateral pretracheal, prelaryngeal, and paraesophageal central compartment neck dissection (CCND).

### 2.2. Postoperative Outcomes

Clinical parameters analyzed included patient characteristics, operative variables, extent of surgery, pathologic findings, and short-term operative outcomes. Pathologic examinations included assessments of disease tumor-node-metastasis (TNM) stage, number of lymph nodes harvested, and number of metastatic lymph nodes. We also assessed perioperative complications, including hematoma, seroma, vocal cord palsy, hypocalcemia, trachea injury, esophageal injury, chyle leakage, and brachial plexus neuropraxia. All patients were followed up in the same manner at the three centers, including clinical examinations within 1 week of discharge and a 3-to-6-month follow up that included a physical examination, neck US, assay of tumor markers (serum thyroglobulin concentration), and/or a ^131^radioactive iodine (^131^RAI) scan. 

### 2.3. Learning Curves

To evaluate the learning curve for robotic versus endoscopic thyroidectomy, we used a protocol similar to those previously described for the evaluation of learning curves for thyroid surgery [5, 9]. That is, we assessed groups of about 100 (96–130) patients who underwent robotic and endoscopic less than total thyroidectomy performed by four surgeons at the time they started performing these operations independently. Each patient was assigned a case number without regard to tumor size or lymph node metastasis. Operation time was defined as the time from first incision to the completion of skin closure and included docking and undocking of the robot. Surgical learning curves were analyzed using a moving average method. The surgical techniques we use for endoscopic and robotic thyroidectomy have been described in detail elsewhere [8, 17–19].

### 2.4. Statistics

Data were analyzed using SPSS 12.0 for Windows (SPSS Inc., Chicago, IL, USA). All data are expressed as means ± standard deviations (SDs), proportions, or absolute numbers. Continuous data were compared using Student's *t*-tests, and categorical data were analyzed using chi-squared or Fisher's exact tests. A *P* value < 0.05 was considered statistically significant. 

A moving average method was used to analyze operation time and learning curves. Creating an average that “moves” with the addition of new data results in “smoothing” of the process being analyzed, thus reducing the effects of fluctuations. We used a moving average of 20 to reduce variations and accentuate trends [20].

## 3. Results

### 3.1. Demographic and Clinical Data

Table 1 shows the demographic data and extent of surgery for the 2,612 patients included in this study. Mean age and gender distribution were equivalent for the robotic and endoscopic groups, but types of operation differed significantly. Less than total thyroidectomy was performed in 693 patients (82.2%) in the endoscopic group and in 1,063 (60.1%) in the robotic group, whereas total thyroidectomy was performed in 150 patients (17.8%) in the endoscopic group and in 706 (39.9%) in the robotic group (*P* = 0.004). Modified radical neck dissection (MRND) was also performed more frequently in the robotic than in the endoscopic group (3.4% versus 1.7%, *P* < 0.001). 

### 3.2. Pathologic Findings and Perioperative Outcomes

Pathologic results are shown in Table 2. There were no significant between differences groups in type of tumor, tumor size, multifocality, and bilaterality. However, the mean number of harvested lymph nodes was significantly higher in the robotic than in the endoscopic group (4.5 ± 2.6 versus 2.9 ± 1.7, *P* < 0.001). More advanced T stage (*P* = 0.011), N stage (*P* = 0.001), and TNM stage (*P* = 0.002) tumors occurred more frequently in the robotic group. All 8 patients with T4a lesions showed local invasion of recurrent laryngeal nerve detected during operation, which could be not be noticed in preoperative imaging study, and underwent Maryland or endoscopic dissector shaving procedure.

Total operation time for subtotal thyroidectomy was similar in the two groups, but was significantly shorter in the robotic than in the endoscopic group for total thyroidectomy (*P* < 0.001). Postoperative hospital stay did not differ significantly (Table 3). Among the perioperative complications observed, transient hypocalcemia and transient hoarseness were the most frequent causes of postoperative morbidity, but there were no between differences groups in incidence. Transient hoarseness resolved in all patients within 6 months, as confirmed by postoperative laryngoscopy, and transient hypocalcemia resolved within 3 months. Transient traction injury from brachial plexus neuropraxia was observed in 3 patients in the robotic and 1 in the endoscopic group. This impairment resulted in pain and movement disorder of the shoulder and upper arm, but resolved spontaneously within 3 months.

Of the 856 patients who underwent bilateral total thyroidectomy, 481 underwent ^131^RAI ablation (range, 30–150 mCi) and a ^131^RAI scan 5–7 days after ^131^RAI ablation. The remaining 375 patients did not undergo ^131^RAI ablation as they were deemed low risk (356 patients) or chose not to undergo such treatment (19 patients). No patient showed abnormal uptake on ^131^I whole body scans. At the time of ^131^RAI ablation (TSH stimulated), mean serum thyroglobulin levels were checked, serum thyroglobulin in 362 (75.2%) was <1 ng/mL and in the remaining 119 (24.8%) was >1 ng/mL (3.1 ± 1.8, range 1.2~9.6 ng/mL). Serum thyroglobulin levels were measured in 1,851 patients at 6–12 months postoperatively (TSH suppressed), with most being maintained at the lowest level (<1 ng/mL). Mean thyroglobulin concentrations did not differ significantly in the robotic and endoscopic groups (0.72 ± 1.84 ng/mL versus 0.61 ± 1.99 ng/mL). Follow-up neck US and neck CT showed recurrence in six patients, 3 in each group, with 3 recurrences in a lateral neck node and 3 in the contralateral thyroid gland. 

### 3.3. Learning Curves for Robotic and Endoscopic Thyroidectomies

**Figure 1:**
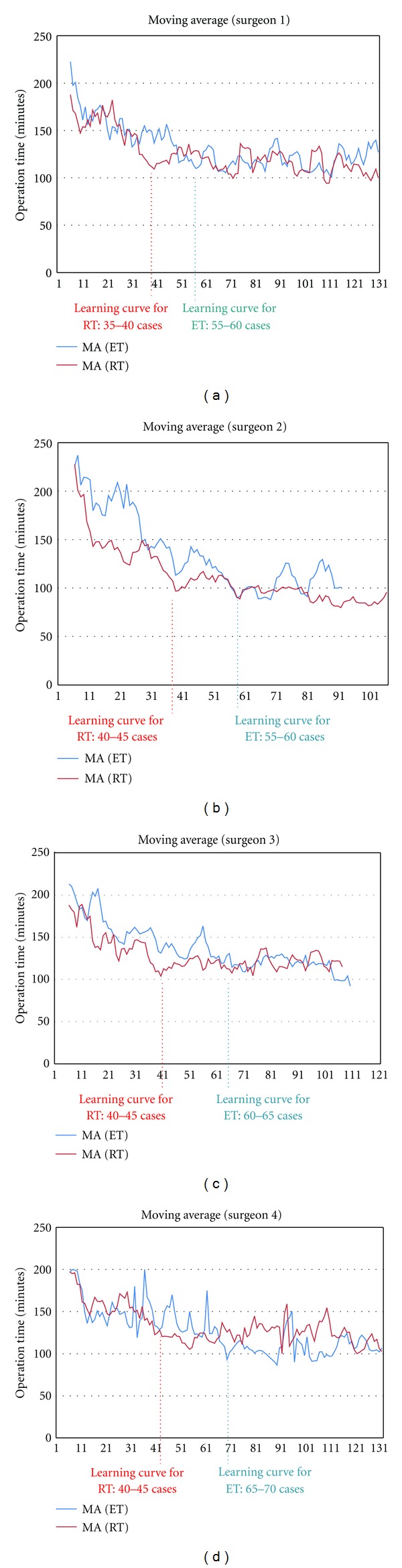
(a–d) Individual learning curves for robotic thyroidectomy (RT) and endoscopic thyroidectomy (ET); The graph plots the time taken to perform each procedure as a function of the number of patients. The moving average method was used to determine changes in operation times for each surgeon. The time required for each surgeon to perform RT decreased after 35–50 patients, whereas the time required by each to perform ET decreased after 55–70 patients.

When we compared the data on about 100 individual patients who underwent robotic and endoscopic less than total thyroidectomy by all involved surgeons, we observed no significant differences in patient-selection criteria. For both procedures, the operation times gradually decreased with accumulating experience. For all 4 surgeons, operation times reached a plateau after 35–45 robotic and 55–70 endoscopic less than total thyroidectomies (Figure 1). 

## 4. Discussion

Robotic thyroidectomy is an emerging technique with early outcomes that are at least comparable to those of conventional endoscopic thyroidectomy, with some end points appearing superior. Our series represents the largest comparison of robotic and endoscopic thyroidectomy to date, with results suggesting comparable operation times, perioperative outcomes, and complications. In addition, early-term oncologic outcomes demonstrated that robotic thyroidectomy resulted in acceptably complete resection and radical dissection, with an extremely low rate of recurrent disease at follow up. Moreover, to our knowledge, this study is the first multicenter trial to compare the learning curves of robotic and endoscopic thyroidectomy. 

### 4.1. Comparison of Perioperative Outcomes with Robotic and Endoscopic Thyroidectomy

The goal of thyroidectomy plus neck dissection in patients with thyroid cancer is the complete surgical removal of the entire thyroid gland, along with radical cervical lymphadenectomy when necessary. Other goals include rapid convalescence and complete preservation of the recurrent and superior laryngeal nerves and the parathyroid gland. Only a few previous large series have compared perioperative outcomes in patients undergoing robotic and endoscopic thyroidectomy [8–11] (Table 4). Our previous experience suggests that robotic thyroidectomy may be associated with decreased operation times and increased harvest of cervical LNs, outcomes related to the operative dexterity of the flexible robotic instruments, and a 3D surgical view [5–9]. In contrast, endoscopic thyroid dissection and lymph node retrieval may be more difficult and time consuming because of the straight angles of the endoscopic instruments and a 2D operative field [1–4]. Another superiority of robotic thyroidectomy over conventional endoscopic thyroidectomy is that surgeons use three arms during operation. In conventional endoscopy, the surgeon can steer only two arms during dissection, and this makes it difficult to create appropriate conditions for dissection. However, in robot technique, the surgeon can handle the Prograsp forceps during using the Maryland dissector, optimal dissection planes can be obtained by applying traction and countertraction to thyroid gland, and could position the thyroid gland to allow fine, dexterous dissection after swapping arms by controlling robotic arms himself [5–8].

A recent retrospective comparison of 580 patients who underwent robotic thyroidectomy and 570 who underwent conventional endoscopic thyroidectomy at a single center found that the operation time was shorter and the mean number of central LNs retrieved greater in the robotic than in the endoscopic group [8]. These findings were also observed in a recent comparison of robotic and endoscopic thyroidectomy performed by a single surgeon [9], providing further evidence that the robotic technique provides better results that conventional endoscopy in thyroid cancer patients [8–11]. Table 4 summarizes the published findings in studies comparing robotic and endoscopic thyroidectomy. Our findings were similar, in that mean operation times were 13.6% shorter (*P* < 0.001) and the numbers of LNs retrieved significantly greater (*P* < 0.001) for robotic than for endoscopic total thyroidectomy. 

Objective comparisons are limited, however, by the lack of long-term surgical outcomes and by nonuniformity in defining, assessing, and reporting postoperative outcomes. Larger prospective studies, with uniform definitions of parameters, methodologies of data collection, and times of assessment are needed to compare operative outcomes of the two procedures. 

### 4.2. Learning Curves for Robotic and Endoscopic Thyroidectomy

The operation time required by a single surgeon to perform robotic thyroidectomy using a gasless transaxillary approach was found to reach a plateau after 40–45 operations [5]. In addition, the learning curve for inexperienced surgeons to perform robotic thyroidectomy was 35–40 operations, whereas that for endoscopic thyroidectomy using a gasless transaxillary approach was 55–60 operations [9]. An extensive review of the outcomes of learning curves for robotic thyroidectomy in the Republic of Korea categorized patients into two groups, those treated by surgeons with and without experience in robotic thyroid surgery [13]. The learning curves for robotic thyroidectomy were 50 for total and 40 for less than total thyroidectomies. Moreover, once through the learning curve period, operation times and perioperative parameters for previously inexperienced surgeons were similar to those of the experienced surgeons, indicating that the former had acquired the necessary technical skills to perform robotic thyroidectomy successfully [13]. Despite different methodologies and variables in different studies, the results of these investigations of surgical learning curves for robotic thyroidectomy showed similar operation time-related results [5, 9, 13, 16] (Table 5). Similar to these studies, we found that the learning curve was significantly shorter for the robotic than for the endoscopic technique. For all 4 enrolled surgeons at 3 centers, the learning curves were found to be 35–45 patients for robotic and 55–70 for endoscopic less than total thyroidectomy. In this study, we could not perform the comparative evaluation for learning curves between robotic and endoscopic total thyroidectomy, because the number of endoscopic total thyroidectomy cases was too low to analyze the learning curve for this procedure.

This study had potential shortcomings. First, although all 4 surgeons in our study used the same technique, all procedures could not be completed with the same quality of manipulations. Second, patients were not randomized, as it was neither ethically nor geographically possible to move patients from one center to another. This was likely a result of selection bias of our patients and our overall experience with endoscopic and robotic surgery. Third, the short history of robotic thyroidectomy makes assertions on the oncologic safety and the surgical learning curve premature. In addition, the surgeon had experience in performing several endoscopic thyroidectomies prior to any robotic thyroidectomies, and this is likely to have influenced comparisons of operation times and learning curves. Therefore, further study is required to confirm these findings and to assess the surgical learning curve for surgeons who have no experience of endoscopic surgery. Moreover, a prospective and randomized study with longer follow-up period should be conducted to evaluate the role of robotic surgery in thyroid disease.

In conclusion, we have shown that the robot technology we used could overcome some of the technical limitations associated with conventional endoscopic procedures, with reduced operation times and increased lymph node retrieval. Moreover, we found that the learning curve for robotic thyroidectomy was shorter than that for endoscopic thyroidectomy. Prospective randomized studies are required to evaluate the actual learning curves of inexperienced endoscopic surgeons for robotic thyroidectomy and lymph node dissection. 

## Figures and Tables

**Table 1:** Demographics and extent of surgery in patients with thyroid carcinoma treated by robotic thyroidectomy versus endoscopic thyroidectomy.

	Robot group (*n* = 1769)	Endoscopy group (*n* = 843)	*P* value
Age, mean (years), range	39.4 ± 9.1 (13–70)	37.5 ± 9.4 (6–50)	NS*
Gender			
Female	1637 (92.5%)	824 (97.7%)	NS
Male	132 (7.5%)	19 (2.3%)	
Extent of surgery			
Less than total	1063 (60.1%)	693 (82.2%)	0.004
Unilateral total	595 (33.6%)	307 (36.4%)	
Unilateral total + partial	177 (10.0%)	208 (24.7%)	
Unilateral total + subtotal	291 (16.4%)	178 (21.1%)	
Total	706 (39.9%)	150 (17.8%)	
Extent of neck node dissection			
No dissection	23 (1.3%)	59 (7.0%)	<0.001
CCND**	1675 (94.7%)	770 (91.3%)	NS
Selective node dissection	11 (0.6%)	0 (0%)	NS
MRND^§^	60 (3.4%)	14 (1.7%)	<0.001

NS*: nonspecific finding, CCND**: central compartment neck dissection, MRND^§^: modified radical neck dissection.

**Table 2:** Comparison of pathologic findings between patients treated with robotic thyroidectomy and endoscopic thyroidectomy.

	Robot group (*n* = 1769)	Endoscopy group (*n* = 843)	*P* value
Pathology			NS
Papillary carcinoma	1758 (99.3%)	837 (99.3%)	
Follicular carcinoma	5 (0.3%)	6 (0.7%)	
Medullary carcinoma	5 (0.3%)		
Hurthle cell carcinoma	1 (0.1%)		
Tumor size, mean (cm)	0.5 ± 0.5	0.4 ± 0.5	NS
Multifocality			NS
Yes	469 (26.5)	110 (13.0%)	
No	1300 (73.5)	733 (87.0%)	
Bilaterality			NS
Yes	208 (11.8%)	72 (8.5%)	
No	1561 (88.2%)	771 (91.5%)	
Mean retrieved central LN (*n*)	4.5 ± 2.6	2.9 ± 1.7	<0.001
Mean metastatic central LN (*n*)	1.2 ± 0.9	1.0 ± 0.7	NS
TNM stage			
T1	905 (51.2%)	513 (60.9%)	0.011
T2	16 (0.9%)	15 (1.8%)	
T3	841 (47.5%)	314 (37.2%)	
T4a	7 (0.4%)	1 (0.1%)	
N0	1131 (64.0%)	605 (71.7%)	0.001
N1a	570 (32.2%)	224 (26.6%)	
N1b	68 (3.8%)	14 (1.7%)	
Stage			0.002
Stage I	1480 (83.7%)	750 (89.0%)	
Stage II	275 (15.5%)	92 (10.9%)	
Stage IVa	14 (0.8%)	1 (0.1%)	

LN: lymph node.

TNM: tumor-node-metastasis.

**Table 3:** Comparison of perioperative outcomes between patients treated with robotic thyroidectomy and endoscopic thyroidectomy.

	Robot group (*n* = 1769)	Endoscopy group (*n* = 843)	*P* value
Total operation time (min), range			
Total thyroidectomy	149.2 ± 32.3	172.7 ± 66.7	<0.001
Subtotal thyroidectomy	122.3 ± 32.4	127.2 ± 41.3	NS
Postoperative hospital stay (days)	3.3 ± 1.3	3.4 ± 1.1	NS
Postoperative complications (*n*)			
Transient hypocalcemia	276/706 (39.1%)	55/150 (36.7%)	NS
Permanent hypocalcemia	0 (0%)	2 (0.2%)	
Transient hoarseness	68 (3.8%)	41 (4.9%)	
Permanent hoarseness	8 (0.5%)	1 (0.1%)	
Flap hematoma	10 (0.6%)	8 (0.9%)	
Observation	8 (0.5%)	5 (0.6%)	
Reoperation	2 (0.1%)	3 (0.4%)	
Seroma	40 (2.3%)	19 (0.3%)	
Tracheal injury	3 (0.2%)	4 (0.5%)	
Esophageal injury	0 (0%)	0 (0%)	
Transient chyle leakage	6 (0.3%)	3 (0.4%)	
Transient traction injury	3 (0.2%)	1 (0.1%)	
*d*/*t* brachial plexus neuropraxia			

**Table 4:** Endoscopic versus robotic thyroidectomy studies. Case series comparing perioperative outcomes.

Author, year	Study design	No. (patients)	Approach	Comparative parameters	Perioperative results	Complication rate	Oncologic safety (surgical completeness such as harvested lymph node)	Other notable findings
Lee et al. (2011) [9]	Prospective, controlled, single surgeon	RT* (163) versus ET^§^ (96)	GT**	Perioperative outcomes, surgical learning curve	Operation time (ET > RT) Learning curve (ET > RT) Advanced cancer (RT > ET)	No difference	Retrieved LN (RT > ET)	First comparative study between RT and ET. Showed superiority of RT in terms of operation time, lymph node retrieval, and learning curve

Lang and Chow (2011) [11]	Retrospective, controlled, single surgeon	RT (7) ET (39)	GT	Perioperative outcomes	Operation time (RT > ET)	No difference	No data	Described initial experience of RT in Hong Kong

Lee et al. (2011) [8]	Retrospective, controlled, single center	RT (580) ET (570)	GT	Perioperative outcomes	Operation time (ET > RT) Advanced cancer (RT > ET)	Transient- hypoparathyroidism (RT > ET)	Retrieved LN (RT > ET)	RT was found to be superior to ET in terms of operation time, and LN retrieval.

Kim et al. (2011) [10]	Retrospective, single center	RT (69) ET (95) OT (138)	BABA^§§^	Perioperative outcomes	Operation time (RT > ET > OT^#^)	No difference	Surgical completeness (RT = OT > ET) Retrieved LN (RT = OT = ET)	First comparative study of RT versus OT by analyzing postoperative outcomes

RT*: robotic thyroidectomy, GT**: gasless transaxillary approach, ET^§^: endoscopic thyroidectomy, BABA^§§^: bilateral axillo-breast approach, OT^#^: open thyroidectomy.

**Table 5:** Published data for surgical learning curves for robotic thyroidectomy.

Author, year	Study design	No. (patients)	Approach	Pathology	Operation	Operation time	Complications (Major)*	Methods for analysis	Surgical learning curve for robotic thyroidectomy
Kang et al. (2009) [5]	Retrospective (single surgeon)	338	GT**	PTC^§^ (332) Benign (6)	TT^§§^ & CCND^#^ (104) LTT^∆^& CCND (234)	Total: 144.0 ± 43.5 Console: 59.1±25.7	5/338 (1.5%)	Moving average	RT^##^ (Console time): 40–45 cases

Lee et al. (2011) [9]	Retrospective (single surgeon)	163	GT	PTC (151) FTC*γ* (1) Benign (11)	TT (48), LTT (115) CCND (149)	Total: 110.1 ± 50.7 Console: 50.9 ± 11.4	2/163 (1.2%)	Moving average	RT: 35-40 cases, ET^∆∆^:50–60 cases

Lee et al. (2011) [13]	Prospective (multicenter study)	644	GT	PTC (616) FTC (13) Benign (15)	TT & CCND (353) LTT & CCND (291)	Total: 181.5 ± 78.2 (≤50^*γγ*^)→141.5 ± 33.0 (>50) Console: 50.9 ± 11.4	2/644 (0.3%)	Comparative analysis between beginners and experience surgeons	RT (LTT): 40 cases RT (TT): 50 cases

Kandil et al. (2012) [16]	Prospective (single surgeon)	100	GT	No data	TT (22) LTT (69) CT^*√*^ (9)	Total: 121.9 ± 63.8 (≤45^*√√*^)→104.2 ± 71.5 (>45) Console: 51.3 ± 31.1 (≤45) →45 ± 36.4 (>45)	1/100 (1.0%)	Comparative analysis between early experience and late experience	RT: 45 cases

Complications (Major)*: major complications mean permanent damages such as recurrent laryngeal nerve injury, permanent hypocalcemia, hematoma of muscle flap need to reoperation, hemorrhage of a major vessel need to reoperation, trachea injury, Honor's syndrome, major chyle leakage, and brachial plexus neuropraxia (not including minor complications such as transient hypocalcemia, transient hoarseness, wound seroma, wound infection, and hematoma of muscle flap only need to conservative management), GT**: gasless transaxillary approach, PTC^§^: papillary thyroid carcinoma, TT^§§^: total thyroidectomy, CCND^#^: central compartment node dissection, RT^##^: robotic thyroidectomy, LTT^∆^: less than total thyroidectomy, ET^∆∆^: endoscopic thyroidectomy, FTC^*γ*^: follicular thyroid carcinoma, ≤50^*γγ*^: experience for robotic thyroidectomy were less than 50 cases, CT^*√*^: completion thyroidectomy, ≤45^*√√*^: experience for robotic thyroidectomy was less than 45 cases.
